# Amino Acids That Centrally Influence Blood Pressure and Regional Blood Flow in Conscious Rats

**DOI:** 10.1155/2012/831759

**Published:** 2012-05-29

**Authors:** Yumi Takemoto

**Affiliations:** Department of Neurophysiology, Graduate School Biomedical Sciences, Hiroshima University, Kasumi-cho 1-2-3, Minami-ku, Hiroshima, 734-8551, Japan

## Abstract

Functional roles of amino acids have increasingly become the focus of research. This paper summarizes amino acids that influence cardiovascular system via the brain of conscious rats. This paper firstly describes why amino acids are selected and outlines how the brain regulates blood pressure and regional blood flow. This section includes a concise history of amino acid neurotransmitters in cardiovascular research and summarizes brain areas where chemical stimulations produce blood pressure changes mainly in anesthetized animals. This is followed by comments about findings regarding several newly examined amino acids with intracisternal stimulation in conscious rats that produce changes in blood pressure. The same pressor or depressor response to central amino acid stimulations can be produced by distinct mechanisms at central and peripheral levels, which will be briefly explained. Thereafter, cardiovascular actions of some of amino acids at the mechanism level will be discussed based upon findings of pharmacological and regional blood flow measurements. Several examined amino acids in addition to the established neurotransmitter amino acids appear to differentially activate brain structures to produce changes in blood pressure and regional blood flows. They may have physiological roles in the healthy brain, but pathological roles in the brain with cerebral vascular diseases such as stroke where the blood-brain barrier is broken.

## 1. Introduction

When the rat spontaneously performs an action such as grooming [1] or walking [2], changes in regional blood flows for head and legs are produced. The brain appropriately regulates blood supply to organs needed for planning of each behavior. For matching cardiovascular demand to each behavior, various kinds of potential neurotransmitters and neuromodulators should work in neuronal networks of the brain relating to the cardiovascular system and behavioral planning. A list of neurotransmitters includes the amino acids glutamate and GABA (gamma-amino-butyric acid) which are well established as endogenously produced excitatory and inhibitory agonists, respectively [3], and appear to play a pivotal role in the central nervous system relating to cardiovascular regulation [4–7]. However, it has been expanding to range the kind and the number of mediators between brain cells from classic neurotransmitter biogenic amines to gaseous neurotransmitters [8] and to gliotransmitters [9]. With respect to amino acids, the concentration of most amino acids in the cerebrospinal fluid is lower than those in the blood [10]. The blood-brain barrier effectively protects the brain from influence of fluctuating concentrations of plasma amino acids [11]. Why are the concentrations of amino acids kept lower in the cerebrospinal fluid? Amino acids other than the established neurotransmitter amino acids may have some functional roles in the brain as is the case with the established ones. The brain contains many nuclei regulating blood pressure and regional blood flow via several pathways [4, 12]. I have hypothesized that some amino acids influence the cardiovascular system through the brain. I have, therefore, begun examining the responses of the cardiovascular system to brain stimulations with amino acids [13, 14]. The anesthesia works at brain level to cause immobility and unconsciousness in animals mainly via GABAa receptors [15], resulting in modified states of the neural networks different from the un-anesthetized state [16]. Therefore, the un-anesthetized freely moving rat has been used in the examinations of brain stimulation with an intracisternal injection of amino acid solution. I will mention findings on several amino acids that modulate the cardiovascular system in this paper, after concisely describing the basic knowledge of central cardiovascular regulation. See references for details: hemodynamics [17, 18], central cardiovascular regulation [12, 19–21], or recent knowledge on amino acids including various functions [22]. In the following sections, I will summarize findings on several amino acids in addition to the established amino acid neurotransmitters (GABA, glycine, and glutamate) that show functional roles in modifying the cardiovascular system. Understanding the mechanisms of how the brain operates is still a great challenge. I would like to suggest that amino acids other than the established amino acid neurotransmitters and/or neuromodulators may have an important role in how the brain functions.

## 2. Why Are Amino Acids the Focus?

My first concern was roles of the blood-brain barrier. One of the roles is to help to maintain lower concentrations of amino acids in the cerebrospinal fluid than in the plasma [10]. Concentrations of L-proline and L-cystine (dimer of L-cysteine) in the cerebrospinal fluid are extremely low [10]. Why has the brain developed to maintain lower values of such amino acids in the cerebrospinal fluid? The brain tissue surrounding the route of the cerebrospinal fluid may be affected by higher concentrations of L-proline or L-cysteine. Central response to an amino acid might give a clue to decipher the neural wiring relating to proper blood flow shift in animal behaviors. This is the reason I have focused on the role of amino acids in the brain for cardiovascular regulation.

Our whole body uses only 20 amino acids to synthesize proteins and peptides, among more than 300 natural amino acids known at present [22]. One of those proteinogenic amino acids, L-glutamate, has been recognized as an endogenous neurotransmitter for excitatory amino acid receptors at synapses of the central nervous system for several decades [23–25]. Other established historical inhibitory amino acid agonists are GABA, and glycine, which is also one of 20 brick amino acids for proteins [25, 26]. Interestingly, evolutionary process utilizes the ubiquitous nutrient amino acids glutamate and glycine in the brain and spinal cord to signal and communicate between neurons with elaborate mechanisms. Extremely high tissue contents of L-glutamate, GABA, and glycine in the central nervous system [10, 27] would suggest a highly evolved control system that uses these amino acids for the basic common wiring plan to maintain homeostasis throughout the body. Research on the effects of amino acids in the central nervous system began in 1952 with a report from Hayashi on the central convulsive action of glutamate [28, 29]. A possible role of neurotransmitters for amino acids was discussed according to neurotransmitter criteria in 1974 by Curtis and Johnston [30]. They mentioned possible neurotransmitter roles of other amino acids in addition to the later established neurotransmitter amino acids.

With respect to cardiovascular control, GABA, glycine, and glutamate have been known to have an effect on blood pressure since 1954 [31–35]. Takahashi's group first reported a depressor effect of intravenously injected GABA in 1955 [31] and later identified acting sites of GABA as the central origin using various approaches such as ganglionic blocking, intracisternal injection, and direct stimulation with topical application of GABA solution on the dorsal medulla of the anesthetized animal [32]. Intracisternal injection of glycine, GABA, taurine, and L-*α*-alanine into ether-anesthetized rats produced a central depressor action [33]. As for a pressor response, intravenous injection of glutamate was reported to raise blood pressure of the dog by Itoga in 1954 [34] and of rabbits later by Takahashi's group [31]. Itoga had already suggested the vasomotor centre of the medulla as the active site for the pressor response to intravenously injected glutamate [34]. Intracisternal injection of glutamate was later examined and found to produce a strong pressor response in dogs [35]. In studies of central cardiovascular regulation, concern of most researchers appears to have focused on excitant amino acids mainly as tools of chemical stimulation for electrical stimulation to evoke excitation of only neurons but not axons related to blood pressure regulation. For example, homocysteic acid [29] has been repeatedly selected to stimulate neurons as a stimulant tool for targeting excitatory receptors [36–38]. Several groups nevertheless have continued to investigate the role of chemical mediators in central cardiovascular regulation [5–7]. In future, information on which neurons related to cardiovascular regulation have which neurotransmitters identified with classical criteria: storage, release, and inactivation, might become available [8].

## 3. Outline of How the Brain Regulates Blood Pressure and Regional Blood Flow

**Figure 1:**
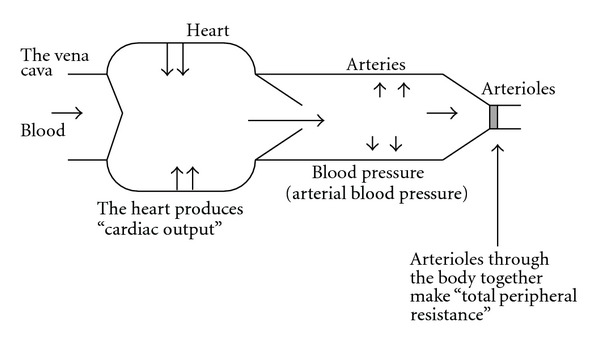
Blood pressure is produced by cardiac output and total peripheral resistance.

**Figure 2:**
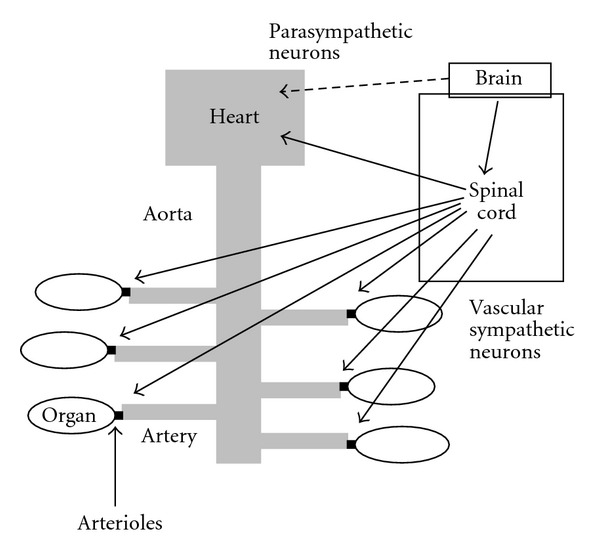
Vascular sympathetic nerves innervate mainly arterioles located just before the vascular bed in each organ. The heart is innervated by both parasympathetic and sympathetic neurons.

**Figure 3:**
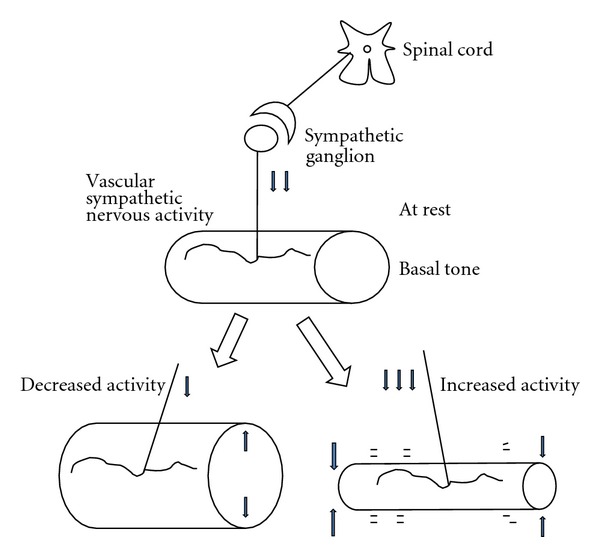
Tonic activities of vascular sympathetic nerves at various levels allow for regulation of flow rate. Basal tone at rest increased by sympathetic nervous activation produces vasoconstriction and a decrease in blood flow rate (an increase in resistance). When basal tone is inactivated, vasodilatation, an increase in flow rate (a decrease in resistance), is produced.

 Blood pressure (specified for arterial blood pressure here) is determined by cardiac output and total peripheral resistance (Figure 1). The heart works spontaneously and propels the blood into the aorta depending on blood volume returned from the vena cava. The brain modifies the cardiac work with cardiac parasympathetic and sympathetic neurons, adrenaline released from the adrenal gland via sympathetic activation, and an increase in venous return with increased sympathetic activity to the capacitance venous vessels. Another variable of peripheral blood flow resistances consists of a lot of acting sites, arterioles all over the body, which are located just before the vascular (capillary) bed of each organ (Figure 2). The brain can modify the size of arterioles with vascular sympathetic neurons and release of several vasoactive factors into the blood stream as mentioned below in detail. For proper delivery of the blood into the demanding organ, regulation of the arterioles could be essential. Vasomotor sympathetic neurons are tonic at rest but regulated by increasing or decreasing activities (Figure 3), resulting in optimal blood flow shift among different vascular beds for demanding organs, in theory.

### 3.1. Brain Has at Least Four Possible Peripheral Pathways for Influencing Arterioles

**Figure 4:**
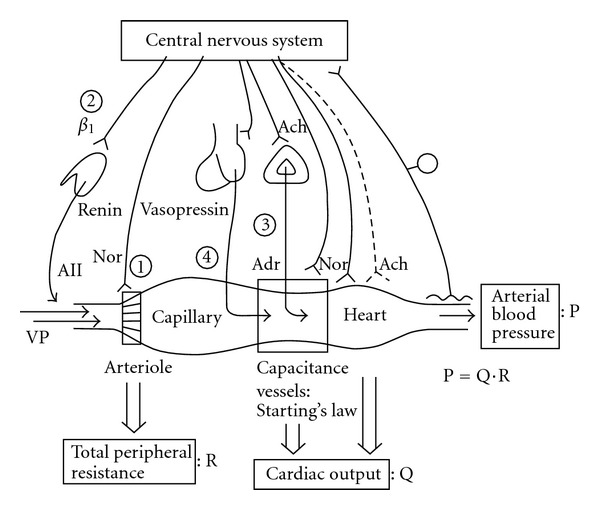
Potential pathways between the central nervous system (CNS) and the cardiovascular system. The CNS regulates the cardiovascular system using various peripheral routs. Arterioles can be regulated by sympathetic neurons and humoral factors of angiotensin II (A II), vasopressin (VP), and adrenaline (Adr), resulting in changes in total peripheral resistance. The heart is regulated by both of parasympathetic and sympathetic neurons and Adr. Capacitance venous vessels are regulated by sympathetic neurons and modify returning blood volume to the heart and cardiac output as predicted by Starling's law. Renal sympathetic neurons can release the enzyme renin from the juxtaglomerular apparatus into the blood via *β*1 adrenoceptors. The renin produces A II via the renin-angiotensin system. The hypothalamus-pituitary system in the forebrain releases VP into the stream. A II and VP constrict arterioles markedly. Adrenal sympathetic neurons release Adr into the blood. The central nervous system monitors arterial blood pressure with visceral afferents terminated in the big arteries. Ach: acetylcholine, Nor: noradrenaline. circles 1–4; see text.

 Stimulation of central nervous system influences arterioles and/or blood pressure through at least four ways (Figure 4) [39]: one is the vasomotor sympathetic neurons in different vascular beds (Figures 2 and 4 circle 1), second is renal sympathetic neurons (Figure 4 circle 2) that release the enzyme renin from the renin-secreting granular cells in the afferent arteriole to finally produce a potent vasoactive peptide, angiotensin II, in the blood via the renin-angiotensin system [40, 41], third is adrenal sympathetic neurons (Figure 4 circle 3) that release adrenaline into the blood [42], and finally the hypothalamus-pituitary system that releases the vasoactive or antidiuretic peptide, vasopressin, in the blood (Figure 4 circle 4) [19]. Central excitatory stimulation of those pathways induces a simple pressor response through vasoconstriction by noradrenaline, angiotensin II, and vasopressin and/or increase in cardiac output with adrenaline, but inhibitory stimulation produces a depressor response where the corresponding tonic pathway at rest is deactivated. Because total vascular tone is mainly maintained by vasomotor sympathetic neurons in the animal with normal blood pressure, responses of regional blood flow resistances to inhibitory stimulation would affect vascular beds which are tonic at rest and responsible for maintaining the normal blood pressure. In the same way, genetically or experimentally produced hypertensive animals could be examined with blockade of the above-mentioned four possible peripheral pathways to elucidate the cause of prolonged hypertensive states.

### 3.2. Brain Nuclei Where Chemical Stimulations Produce Changes in Blood Pressure

 Chemical stimulation of the brain has been used to produce changes in blood pressure, because they are expected to stimulate only the cell body of the neuron [44]. As stimulants, ionotropic excitatory amino acid receptor agonists, L-glutamate, DL-homocysteic acid, and kainic acid, have been chosen for most cases, but other chemicals such as serotonin, acetylcholine, GABA, glycine, angiotensin II, endothelin, and receptor agonists and antagonists have also been tested.

**Figure 5:**
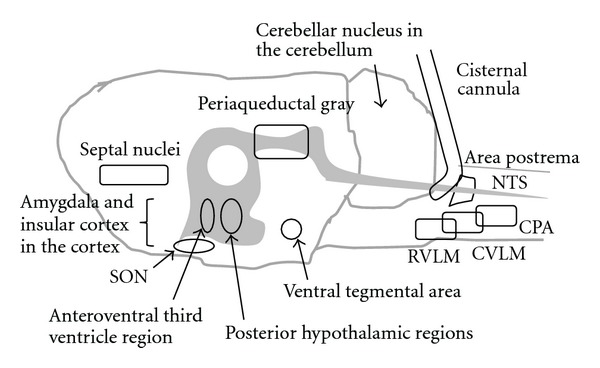
Potential brain nuclei of which neurons respond to intracisternal injection of amino acid solution, in the sagittal view of the rat brain. SON, the supraoptic nucleus of the hypothalamus; NTS, the nucleus tractus solitarii; RVLM, rostral ventrolateral medulla; CVLM, caudal ventrolateral medulla; CPA, caudal pressor area. Gray area indicates the ventricular system from the third ventricle to the central canal in the spinal cord.

 There are several well-examined neuronal groups which change blood pressure at medulla level when chemically stimulated (Figure 5); rostral ventrolateral medulla (RVLM) [16, 45–49], caudal ventrolateral medulla (CVLM) [47, 50–56], and nucleus tractus solitarii (NTS) [57–64]. Neural pathways of a reflex which detects changes in blood pressure and returns it to the original level via mainly sympathetic neurons (called the baroreceptor reflex) include NTS, RVLM, and CVLM. Baroreceptor afferent neurons of vagal and glossopharyngeal nerves terminate second-order neurons in the NTS [65–67] that send information to GABAergic interneurons in the CVLM [68]. When baroreceptors in the aortic arch and carotid sinus detect an increase in blood pressure, excited GABAergic neurons in the CVLM inhibit presympathetic neurons in the RVLM, leading to a decrease in sympathetic activity and resulting in restoration of original blood pressure [21]. RVLM is believed to contain the most essential neuronal group to maintain the resting and tonic vasomotor sympathetic activity, since lesion or inhibition of RVLM produces severe hypotension equivalent to a spinal transection [50, 69] and blockade of many cardiovascular-related reflexes [12].

Other parts of the brain that produce changes in blood pressure with chemical stimulation include caudal pressor area (CPA) (Figure 5) [70], area postrema [71], rostral ventromedial medulla [72–74], caudal raphe nuclei [75–79], nucleus ambiguous [80], cerebellar sublobule IX (lateral and medial uvula) [81–85], and the fastigial nucleus [4, 44, 86–89] at the medulla and cerebellum. The others are A5 noradrenergic cell group [90–92], locus coeruleus (A6 group) [93, 94], parabrachial area [95–100] in the pons, ventral tegmental area [101–103] and periaqueductal gray matter [104–106] at the midbrain level, and lateral and posterior hypothalamic regions [105, 107–110], amygdala [111, 112], septal nuclei [113], and insular cortex [114, 115] at the forebrain level. Vasopressin-containing neurons in the supraoptic nucleus and paraventricular nucleus of the hypothalamus are also responsible for the pressor response to chemical stimulation via vasopressin release even in awake rats [116, 117].

Neural networks made from the above-mentioned neuronal groups and yet unknown ones could regulate blood pressure and regional blood flow to the vascular bed, corresponding to various needs of the body. Synapses in the neuronal networks should have a lot of combinations of endogenous agonists and receptors like those in autonomic nerves [118], sympathetic preganglionic neurons [119], and medullary presympathetic neurons [7]. A lot of work has been devoted to finding the mechanisms for the appropriate responses to complicated body needs with expanding lists of neurotransmitters and their receptors related to cardiovascular regulation [4, 6, 7, 12, 119]. However, the details of in vivo body function at the synaptic level remain poorly understood.

## 4. Potential Diffused Parts of the Brain with Intracisternal Approach

 Intracisternal injection of chemical solution has several merits for surveying central effects of a lot of amino acids. First, limited areas of the brain around the cisterna magna could be stimulated (Figure 5). The cerebrospinal fluid flows from the lateral ventricles to the cisterna magna through the third ventricle, the aqueduct, and the fourth ventricle, with weak positive pressure [10]. Potential diffused regions with intracisternal injections in the freely moving rat are the ventral medulla surface, excluding part of the dorsal brainstem, and the surface areas around the flow route of the cerebrospinal fluid [120]. The flow route includes the RVLM, CVLM, and CPA located in the ventral surface of the medulla and the NTS located in the dorsal surface of the medulla. The NTS neurons receive afferent information from the body [121] and contribute to keep blood pressure stable via mainly the CVLM then to the RVLM in the baroreceptor reflex [12, 21]. The CPA neurons appear to be included for maintenance of blood pressure but via the RVLM and/or CVLM [21]. Therefore, the RVLM and CVLM would have the greatest influence over changes in blood pressure with intracisternal stimulation of amino acids, as the final stations of the possible network. I have established the intracisternal injection method using polyethylene tubing that produces a minimal lesion of the brain, differently from a stainless steel tube. It was originally developed to take cerebrospinal fluid from freely moving rats [122, 123]. For injection of amino acid solution, the tip was inversely oriented from a caudal side to a rostral side. With respect to acting sites, my recent studies with c-Fos expression [124] and another study using a radioisotope [125] suggest the supraoptic vasopressin-containing nuclei situated in the ventral surface area of the brain in addition to RVLM and CVLM.

## 5. Amino Acids with a Pressor Action

 In the first experiment [13], while blood pressure and heart rate were observed, the following amino acid solutions were intracisternally injected in the freely moving rat: L-glutamic acid (59 nmol in 10 *μ*L artificial cerebrospinal fluid, 5.9 mM), taurine (5 *μ*mol), L-proline (10 *μ*mol), L-*α*-alanine (10 *μ*mol), GABA (10 *μ*mol), L-aspartic acid (0.3 *μ*mol), L-valine (7.6 *μ*mol), L-serine (10 *μ*mol), L-methionine (1 *μ*mol), L-isoleucine (3 *μ*mol), L-leucine (1 *μ*mol), L-tyrosine (25 nmol), L-histidine (5 *μ*mol), L-lysine (10 *μ*mol), L-arginine (10 *μ*mol), L-tryptophan (56 nmol), L-asparagine (1.4 *μ*mol), L-glutamine (2 *μ*mol), glycine (10 *μ*mol), L-phenylalanine (0.18 *μ*mol), and L-cysteine (10 *μ*mol). Each dose depended on the solubility but was below 10 *μ*mol in 10 *μ*L. In the later detailed experiments, L-glutamic acid monosodium salt (0.2 M) was used for improvement of solubility [126], the concentration of L-cysteine was lowered to 0.2 M from 1 M due to its strong spasm causing effect [126], and the pH of basic L-arginine solution was adjusted to 7.4 [127]. To examine the stereoselectivity of the pressor response to L-arginine and L-proline, D-arginine [128] and D-proline [129] were injected in other experiments.

 Intracisternal injections of the following amino acids produced pressor responses in the freely moving rat: L-proline, L-arginine, D-arginine, L-cysteine, L-glutamate, L-aspartic acid, and L-asparagine [13, 126–129]. The pressor responses were dose dependent, became maximal between 1 min and 10 min after injections, and returned to normal within 60 min at longest for all except D-arginine. An increase in heart rate was marked between 1 and 5 min after injection of L-cysteine, and a bradycardiac response was obtained 5 min after injection of L-proline [13].

 An examination of stereoselectivity for the L-arginine response revealed unexpectedly a pressor response to D-arginine injected into the cisterna magna [128]. Because of a possible role of the enzymatic substrate L-arginine for nitric oxide [130], no response to D-arginine stimulation was predicted. Other possibilities as substrates are for kyotorphin (L-tyrosyl-L-arginine) [131] and agmatine [132]. The pressor responses common to both L-arginine and D-arginine apparently denied the possibility as a substrate of L-arginine for the converted different active principles, because of strict stereospecificity of the enzymes. However, as mentioned below, each mechanism for the pressor response was distinct. Active principles of L-arginine and D-arginine for their cardiovascular responses remain puzzling.

 A weak depressor response to D-proline was obtained [129]. Therefore, it is suggested that the neurotransmitter candidate L-proline [133] itself acts on some receptors in the neuronal network relating to cardiovascular regulation.

## 6. Amino Acids with a Depressor Action

 Intracisternal injection of the following amino acids produced depressor responses in the freely moving rat: L-serine, L-sarcosine (N-methyl-glycine), L-*α*-alanine, L-*β*-alanine, taurine, GABA, and glycine [14, 43, 134, 135]. The concentration was 1 M for all except taurine which was 0.5 M.

The depressor and bradycardiac responses were at max between 5 min and 30 min after injections, and blood pressure returned to the original level 60 min or later. The depressor response to L-serine was occasionally accompanied with transient pressor period between 5 and 10 min [14, 134], differently from other depressor amino acids. The mechanism of L-serine to induce two-phase changes in blood pressure could be different from others. Of these depressor amino acids, L-serine, L-*α*-alanine, and L-*β*-alanine have been introduced as electrophysiologically depressant amino acids related structurally to GABA, glycine, and taurine by Curtis and Johnston [30]. Then, L-*β*-alanine is suggested to be a neurotransmitter at present [136].

## 7. Regional Blood Flow Changes with Several Amino Acids of the Pressor Action

**Figure 6:**
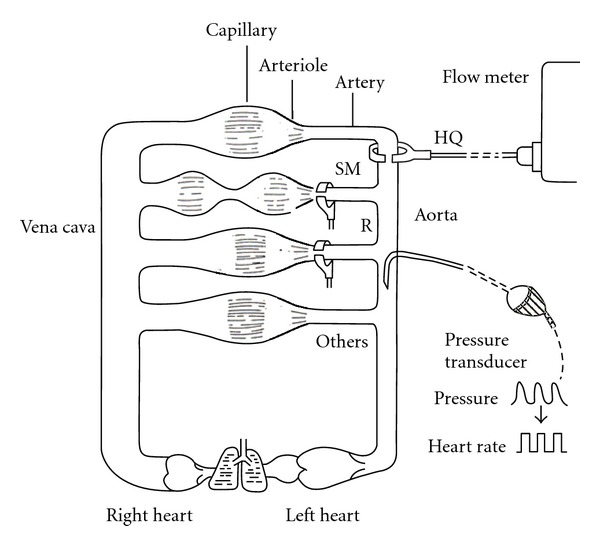
Regional blood flow measured in three arteries after intracisternal injection of L-proline, L-arginine, or D-arginine. SM, superior mesenteric artery; R, renal artery; HQ, hindquarter or the terminal aorta. Each experiment observed the flow in one of three arteries with arterial blood pressure and heart rate in the freely moving rat.

 Regional blood flow measurement in three arteries (Figure 6) was performed for L-proline, L-arginine, and D-arginine. Information on changes in regional blood flow will give a clue to decipher how an amino acid modifies regional peripheral resistance and changes blood pressure.

**Figure 7:**
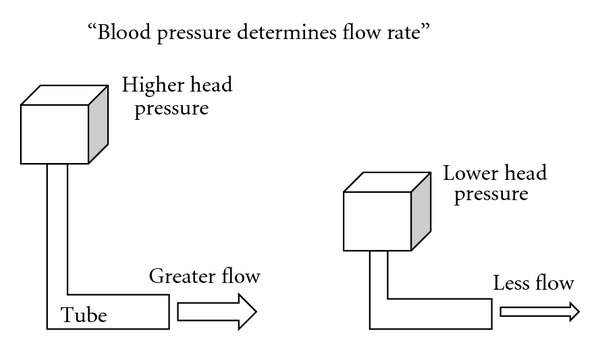
Higher head pressure produces greater flow when the tube keeps the same diameter without any influence.

**Figure 8:**
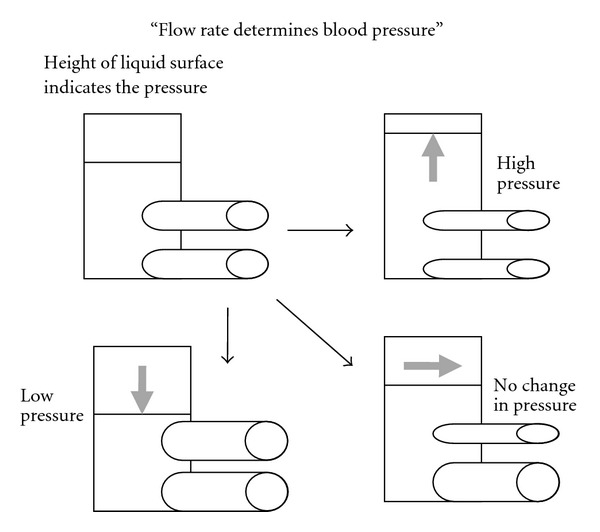
The pressure is increased in constricted tubes but lowered in dilated tubes and unchanged for a combination of constriction and dilatation.

Before seeing the hemodynamic data, explanation of flow measurement and relationship among regional blood flow, regional blood flow resistance, and blood pressure would be needed. An electromagnetic flow probe set around the artery monitors the flow volume rate (not velocity) in the freely moving rat [137] (Figure 6). The blood flow rate (volume/min/100 g weight) measured in an artery just before the vascular bed of an organ reflects the net changes in sizes of arterioles within the organ. Namely, stronger arteriolar dilatation produces more arterial blood flow, but stronger vasoconstriction produces less flow. At rest, the size of arteriole is mainly determined by the basal tone of vascular sympathetic neurons (Figure 3). When the vascular sympathetic discharge is increased, the resultant vasoconstriction reduces flow to the corresponding artery. When arterioles in a particular vascular bed are not under the control of sympathetic neurons, higher blood pressure produces blood flow increase. Namely, when tube (artery) size is constant and head (blood) pressure is different, higher head pressure produces greater flow (Figure 7). Therefore, to take the influence of blood pressure changes into account, blood flow resistance or conductance is used to evaluate the net effect of the vascular bed. The studies have expressed blood flow resistance or vascular resistance that is blood pressure divided by flow. Changes in blood pressure always influence blood flow, but changes in blood flow can make changes in blood pressure when they influence the total peripheral flow resistance that is the net change in all the vascular beds. When liquid in a tank flows out from the exits, constricted tubes together produce high back pressure, but dilated tubes together produce low back pressure, and a combination of constricted and dilated tubes results in no change in back pressure (Figure 8). The final example shows what happened when the rat walked spontaneously. The flow was shifted from carotid artery to hindquarters (aortic terminal for legs) without changes in blood pressure that was also supported with an increase in cardiac output [2]. However, grooming behavior increased blood pressure slightly (by 10 mmHg) with blood shift in the opposite direction [1]. Here, we see changes in regional blood flow resistance when amino acids produce the pressor response.

 Intracisternal injection of L-proline (1 M) produced a threefold increase in blood flow resistance in the superior mesenteric artery, double in renal artery, and no significant change in hindquarters [129]. With respect to each original blood flow with the pressor change (25%), superior mesenteric flow was decreased but there was no significant change in either renal flow or hindquarters flow [129]. If the pressor change is not considered, it appeared that vasoconstriction only in the superior vascular bed contributed to the pressor response. However, the resistance data indicate that L-proline stimulation of the brain produced strong superior mesenteric and minor renal vasoconstriction, resulting in the pressor response.

 In the case of L-arginine (0.5 M), vasoconstriction in the superior mesenteric (80%) and renal arteries (60%) was almost equivalent, but hindquarters resistance was unchanged for 25% increase in blood pressure, suggesting an equivalent contribution of the splanchnic vasoconstriction to the pressor response [127].

 The original aim of D-arginine injected into the cisterna magna was to examine the stereospecific effect of L-arginine, as mentioned above. However, the results indicated that pressor response to D-arginine was the same as L-arginine, but distinct changes in regional blood flows were observed. There were almost equivalent increases in superior mesenteric (60%) and renal resistances (45%) but a decrease in hindquarters resistance (35%) and an increase in total peripheral blood flow or cardiac output by calculation (23%), for the pressor response (25%) to D-arginine (1 M) [128]. The data suggested the major contribution of cardiac output with the minor one of total peripheral vascular resistance to the pressor response of D-arginine, differently from L-arginine. Each amino acid appeared to stimulate different brain nuclei relating to the cardiovascular regulation, resulting in the common pressor response.

## 8. Regional Blood Flow Changes with Several Amino Acids of the Depressor Action

 Regional blood flow measurement in three arteries (Figure 6) was performed for GABA, L-*β*-alanine, and glycine, because of the similarity of the molecular structure [43, 135]. Three amino acids in common decreased hindquarters resistance alone without significant changes in superior mesenteric resistance or renal resistance [135].

**Figure 9:**
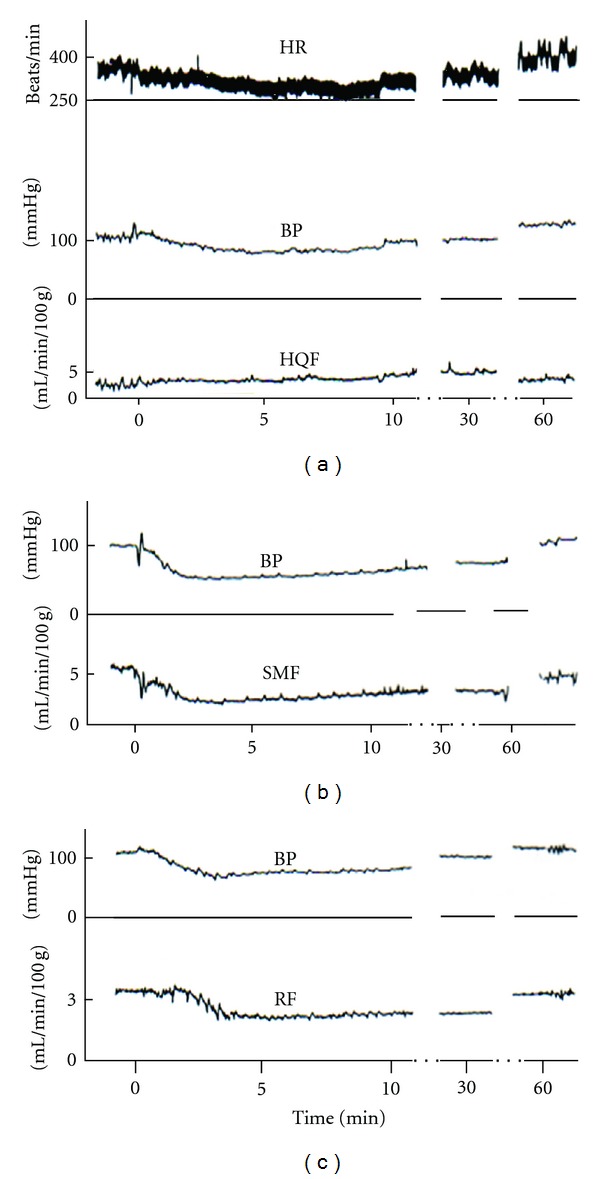
Simultaneous recordings of parameters of circulation of conscious rats in cisternal injection of GABA. HR, heart rate; BP, arterial blood pressure; HQF, hindquarter blood flow; SMF, superior mesenteric blood flow; RF, renal blood flow. Ten micromoles of GABA was injected at 0 in the time scale. From Figure  1 of [43]. (Reprinted with permission from the Physiological Society of Japan).

Figure 9 shows an example of blood flow recordings in three arteries with depressor and bradycardiac responses to intracisternal injection of GABA [43]. The flow changes could be confusing, because hindquarters flow showed no change, but both superior and renal flow decreased almost in parallel to lowering blood pressure. It might be interesting to see autoregulation of renal artery to keep flow constant to some extent during initiating lowering blood pressure, compared with the exact parallel changes of superior mesenteric flow and blood pressure (Figure 9). It is the case that, when the vascular bed has no influence, flow is decreased depending on blood pressure lowering (Figure 7). GABA in the brain could inhibit the tonic resistance in the hindquarters vascular bed alone to reduce blood pressure, along with a bradycardia (probably cardiac output reduction). It appears that hindquarters resistance is regulated by glycine receptors and L-*β*-alanine-sensitive receptors in addition to GABA receptors.

Further detailed evaluation of vascular resistance responses to intracisternal GABA stimulation suggested that resting tone in the carotid in addition to hindquarters vascular beds but not the superior mesenteric, celiac, and renal vascular beds influenced by exogenously applied GABA possibly through GABA receptors not occupied with endogenous GABA [138, 139]. The grooming and walking behaviors produced blood shift between carotid artery and hindquarters [1, 2]. Receptors for GABA and other depressor amino acids may be involved in a program of neuronal networks for producing the proper blood flow shift during a behavior.

## 9. Peripheral Pathways Activated by Several Amino Acids with the Pressor Action

 The pressor response to central amino acids could be produced by increases in total peripheral resistance and/or cardiac output. Vessels and heart are influenced by humoral substances and neural activation via the brain (Figure 4). When the pressor response is observed, we can differentiate possible pathways pharmacologically. Autonomic ganglionic blockade, receptor antagonists, and enzyme inhibitors have been effectively used for the pressor response to an intracisternally injected amino acid.

Regional hemodynamic behaviors responding to venous infusions of vasoactive agonists (vasopressin, angiotensin II, noradrenaline, and adrenaline), which are possibly released into the blood, are also useful to estimate central pathways activated by an amino acid (Figure 4) [39]. Exogenously infused vasopressin dose-dependently increased resistances of all the vascular beds investigated except the celiac vascular bed. Celiac resistance was unchanged while the other four resistances were increased by increasing doses of vasopressin, and it is abruptly increased only at the highest dose when blood pressure increased by 50%. Angiotensin II and noradrenaline infusions produced in common various levels of vasoconstriction in four arteries other than the hindquarters. Both vasoactive substances resulted in unchanged hindquarters resistance, and the order of actions among the other four arteries was distinctly different. For 20% increase in blood pressure, the most potent vasoconstriction was obtained in renal and celiac arteries with noradrenaline infusion and in renal artery with angiotensin II. Adrenaline infusion produced quite different hemodynamic changes. As expected from *β*-receptor stimulation for vasodilatation, hindquarter resistance was decreased in a wide range of adrenaline doses, but without increase in blood pressure in low doses. Surprisingly, a celiac resistance increase compensated for the lowered hindquarters resistance, maintaining blood pressure. Higher doses of adrenaline produced vasoconstriction of other renal and carotid arteries, especially of superior mesenteric artery.

 With pharmacological data and regional vascular responses to circulating vasoactive substances, the distinct central pathways responding to each pressor amino acid are estimated. Ganglionic blockade effectively inhibited the pressor response to L-arginine [127] and D-arginine [128], suggesting that both amino acids could stimulate central pathways relating to autonomic neurons. Vasodilatation in hindquarters produced by D-arginine was attenuated by a *β*-receptor inhibitor [128]. Taking hemodynamic data into account, it is suggested that L-arginine would activate central neurons relating to vascular sympathetic neurons in the superior and renal vascular beds. Because adrenaline could be released with the intracisternal injection, D-arginine appears to stimulate nuclei relating to adrenal sympathetic neurons.

 With respect to L-cysteine [126], L-glutamate [126], and L-proline [129], ganglionic blockade augmented the pressor response. Additional intravenous injection of vasopressin V1 receptor antagonist, however, completely abolished the augmented pressor response to those amino acids. The data suggest involvement of vasopressin release with amino acids. Previous intravenous injection of vasopressin V1 receptor antagonist alone, without ganglionic blockade, significantly attenuated the pressor response to L-proline and L-glutamate but not to L-cysteine. Namely, intracisternally injected three amino acids are suggested to stimulate nuclei relating to vasopressin containing neurons as well as autonomic neurons. Pharmacological data suggest major involvement of vasopressin release and minor roles of autonomic neurons in the pressor response to applications of L-proline and L-glutamate but major involvement of autonomic nervous activation and minor role of vasopressin release in response to L-cysteine.

Intracisternal injection of L-proline likely released vasopressin but unchanged hindquarter resistance [129], although exogenously infused vasopressin produced strong vasoconstriction of hindquarters in a dose-dependent manner [39]. One explanation is that the dilatation additionally produced by adrenaline possibly released with L-proline stimulation counters the vasoconstrictor action of vasopressin, resulting in no change in resistance.

 The findings indicate that each pressor amino acid listed here activates differential central nuclei relating to peripheral pathways of vascular sympathetic neurons, adrenal sympathetic neurons, and vasopressin release via hypothalamus-pituitary route. The amino acids other than D-arginine are nonessential amino acids that can be endogenously synthesized by enzymes for corresponding amino acids in all cells. L-Glutamate is the excitatory neurotransmitter, L-proline is a neurotransmitter candidate [140], L-cysteine could be a neuromodulator [141], and L-arginine, a possible precursor for nitric oxide, kyotorphin, and/or agmatine as above mentioned, is neuroactive.

 These non-essential amino acids can be detective in all cells including neurons for protein synthesis. Therefore, it is not easy to identify which neurons contain which neurotransmitter amino acids. Of them, L-glutamate content in the brain tissue is known to be quite high, but it is a multiplayer for metabolism too. A specific transporter, vesicular glutamate transporter 2, for vesicular packing of L-glutamate in the neuronal terminal has been used to be a good marker of glutamatergic neurons to discriminate the transmitter function of glutamate from metabolite pool [142]. However, even vesicular glutamate transporter 2 coexists with neurotransmitters other than L-glutamate in several neurons [143], becoming a complex situation to clearly delineate a fixed marker of glutamatergic neurons. The localization of neurons containing specific neurotransmitter amino acids appears to need more efforts to be identified.

## 10. Receptors at Central Level in the Pressor Response

 What receptors at central level are involved in the pressor response to L-glutamate, L-proline, and L-arginine injected into the cisterna magna of the freely moving rat? The pressor response to L-proline was blocked by coinjection of a broad spectrum antagonist of ionotropic amino acid receptors kynurenic acid, but the response to L-glutamate and L-arginine remained the same [144]. At least, the central receptor involved in the pressor response to L-proline injected into the cisterna magna could be ionotropic excitatory amino acid receptors.

## 11. Possible Pathological Roles of Amino Acids in the Stroke

 So far, I have mentioned the differential effects of several amino acids directly applied to the brain on the cardiovascular system. The amino acid concentrations in the cerebrospinal fluid will return to and remain low with the help of the active role of the blood-brain barrier in the health [11]. However, there are several reasons the blood-brain barrier can be disrupted including clinical situations such as cerebrovascular diseases of ischemic stroke and intracerebral hemorrhage [145]. Recent studies have revealed that the connection of the barrier can be broken by a molecular cascade activated after ischemia, resulting in vasogenic edema and cell death [145]. With respect to amino acids, pathological amounts of L-glutamate are known to be released into extracellular space in brain ischemia and probably involved in toxic and lethal actions on neurons [146]. The acute cerebrovascular diseases frequently present with mild to moderate spontaneous elevations in blood pressure or acute hypertension [147]. Unlike chronic hypertension as a cause for the diseases, this accompanied hypertension is believed to be a natural process to maintain the blood flow for survived regions. The chemical disturbance produced by the ischemia might stimulate the nuclei responsible for vascular sympathetic activation to result in acute hypertension [148]. After opening of the blood-brain barrier, some amino acids leaked into extracellular space may be involved in additional chemical disturbance to stimulate neurons responsible for blood pressure regulation. Amino acids like L-cysteine derived after brain ischemia [149] might be additional substances causing cell death in addition to acute hypertension.

## 12. Conclusions

 With respect to amino acids for which concentrations in the cerebrospinal fluid are lower than in the plasma, several amino acids among exogenously applied ones showed cardiovascular effects via central mechanisms, as my expectation or hypothesis that amino acids might have some physiological roles in the brain. The clue to initiate the current “amino acids investigation” was the timely blood flow shift in the rat while grooming or walking, and the examinations resulted in a list of additional neurotransmitter and/or neuromodulator candidates relating to the central cardiovascular regulation in freely moving rats. Several examined amino acids with pressor or depressor action differentially influenced regional blood flow and central pathways. There is still much work to be done to decipher how the brain controls blood flow shift for demanding organs. In order to develop hypotheses for future studies, more regional blood flow data together with nervous activity might be useful at the in vivo level. Determination of active sites in the brain responsible for the cardiovascular response to stimulation with each amino acid would be the most desirable for design of further experiments.

 The current paper mainly discussed effects with proteinogenic amino acids on cardiovascular regulation. Mammalian cells can produce plentiful nonproteinogenic amino acids such as L-homocysteine by the related enzymes via several metabolic pathways. A survey of such amino acids for central cardiovascular regulation may provide additional possibilities to find new members for a list of mediators between brain cells or other substances responsible for producing chemical disturbance in brain diseases with disruption of the blood-brain barrier.
